# That Barbaric “Key”

**Published:** 1890-11

**Authors:** E. Cox

**Affiliations:** Auckland, N. Z.


### That Barbaric "Key."

BY E. COX, L.D.S., AUCKLAND, N. Z.

The question whether the "Key" is, or is not, in some cases the suitable instrument will and ought to be determined by experiment. As a rule it should be allowed, I think, to rest in peace having had its day, and accomplished its useful, and in some instances unfortunate work. But I have recently had two cases in which I found in it a last yet lucky resort.

The first was that of a strong, rawboned, young fellow of four and twenty, with capacious mouth and large formidable teeth.

He came with a left lower first molar broken down below the neck over the posterior fang and extensively hollowed over the anterior. It proved a veritable stonewall—fixed, and apparently immovable. Under the forceps what remained of the crown broke away, and the combined fangs defied all further assault by forceps or elevator. A slight coign of vantage was discovered on the lingual front of the anterior fang. I bethought me of the ancient key. Placing a small square of amadou on the buccal side of the alveolar process and carefully applying the fulcrum and claw I turned over the stalwart foe until it lay waiting to be dislodged and removed by forceps.

The second case was one of the most vexatious. A largely built man of almost giant height. A broken down lower left second molar protected and concealed by an elongated first molar. This "ragged rascal" defied and held his ground against all comers and was at last left not in peace but in the condition of the wicked.

Considerable inflammation and suppuration supervened and in the course of a week lancing became necessary and gave considerable relief.

About two months after the first visit the said "rascal" appeared with tempting coolness; his time had come. I should say that not the least trying feature of this case was that the patient was a confirmed, fidgety, and highly nervous bachelor who had known little of pain or illness. He seemed impelled to call at all hours impatiently urging attention, presenting a long melancholy face as though in a doomed if not in a dying state. There is no doubt that want of sleep and the operation had for the time disturbed and irritated his nervous system and it is not surprising that the result upon an excitable temperament like his was "extraction on the brain." After an interval of nine weeks, seeing a chance I gently yet firmly persuaded him to allow a final attempt. Of course, like a timid child, he must first see and understand all about my last resort, and so after full and coaxing explanation, I pursued the same process as in the first instance, and with more complete success, the lurking stowaway leaping right out as if really glad of a change of situation.

The inflammation has since gradually subsided, but the patient has repeated his visits still somewhat distressful in mien and incredulous of cure until the patience of the operator suddenly gave out followed by the speedy exit of his too frequent and tiresom visitor.

